# Inefficacy of different strategies to improve guideline awareness – 5-year follow-up of the hypertension evaluation project (HEP)

**DOI:** 10.1186/1745-6215-9-39

**Published:** 2008-06-25

**Authors:** Jens Hagemeister, Christian A Schneider, Holger Diedrichs, Diana Mebus, Holger Pfaff, Gernot Wassmer, Hans W Höpp

**Affiliations:** 1 Department of Medicine III, University of Cologne, D-50924 Cologne, Germany; 2 Institute and Policlinic of Occupational and Social Medicine, Division of Medical Sociology & Center for Health Services Research Cologne (CHSRC), University of Cologne, D-50924 Cologne, Germany; 3 Institute of Medical Statistics, Informatics and Epidemiology, University of Cologne, D-50924 Cologne, Germany

## Abstract

**Background:**

In spite of numerous guidelines for evidence based diagnostic and therapy adequate knowledge of current recommendations is disappointingly low. In the Hypertension Evaluation Project (HEP I) we showed that awareness of national hypertension guidelines under German practitioners was less than 25% in the year 2000. This indicates the need for efficient strategies to relevantly improve guideline awareness.

**Methods:**

To asses different tools for amending guideline knowledge we used three strategies (guideline in print, interactive guideline, expert seminars) to train 8325 randomised physicians, who had participated in the HEP I trial. Guideline knowledge of the trained physicians was again tested with the HEP questionnaire and compared to a control group of HEP I physicians.

**Results:**

The return rate of questionnaires was 57.9% without a significant distinction between the groups. Overall guideline awareness was still low but remarkably improved compared to the results of HEP I (37.1% vs. 23.7%, p < 0.0001). There was no difference between the trained physicians and the control group (35.8% and 35.9% vs. 39.7%, p = n.s.).

**Conclusion:**

We investigated the influence of different strategies to improve guideline awareness among German physicians. None of our interventions (guideline in print, interactive guideline, expert seminars) brought a notable benefit compared to control group. However, overall knowledge of guideline contents increased from 23.7% to 37.1% over five years. Therefore, other probably multimodal interventions are necessary to significantly improve guideline awareness beyond spontaneous advancement.

**Trial Registration:**

ISRCTN53383289

## Background

Guidelines are recognized as reasonable and necessary decision guidance to guarantee the highest quality level for medical care [1]. Hence, many guidelines for various medical areas have been developed in the last years. In the implementation of guidelines many interface problems can occur, for example problems in the dissemination, the recognition, the acceptance and the practical transformation. Therefore we formerly assessed the knowledge of hypertension guidelines with a survey in a nationwide population of physicians in private practice in Germany in the year 2000. This survey showed that less than 25% of the physicians had adequate knowledge of current recommendations for diagnosis and treatment [2].

An important step for the improvement of the implementation of guidelines is an optimized dissemination of the recommendations within the physician community. The dissemination can be achieved in traditional (e.g. publication, short guideline, seminars) or in innovative (e.g. interactive software, internet) ways. The more traditional ways for information and implementation -publications or hand outs- seem to be ineffective in the light of the above data. Therefore, the goal of the present study was a systematic comparison of traditional and innovative ways for implementation of guideline knowledge for diagnosis and treatment of hypertension. The data collection was performed as a longitudinal study with a follow up duration of five years and was based on the study population of the Hypertension Evaluation Project (HEP I) of the year 2000.

## Methods

### Intervention groups

There were 11 547 general practitioners, internists and cardiologists whose data were kwown from the hypertension evaluation project (HEP I). A total of 8325 participants from this project were divided at random into three intervention groups and one control group (see Table 1).

**Table 1:** Order of study for intervention and re-evaluation of guideline awareness.

**Intervention group**	**Random sample (n = 8325)**	**Executed (n = 6027)**	**Questionnaire (n = 4500)**	**Response (rate) (n = 2474)**
**Seminar**	3825	1527	0	0
**Interactive guideline**	1500	1500	1500	852 (59.8%)
**Printed guideline**	1500	1500	1500	783 (54.9%)
**Control**	1500	1500	1500	839 (58.9%)

One intervention group (projected size n = 3825) was invited to a standardized education seminar. The seminar consisted of a casuistic based lecture of 60 minutes and a discussion of 30 minutes. The seminars were planned in Bremen, Dresden, Düsseldorf, Hannover und Karlsruhe. All study participants within a radius of 50 km were invited to the seminar. The number of participants was calculated with 20% of the invited physicians.

The printed summary of the hypertension guideline of the German Hypertension Society (edition of 2003) was sent to a randomized group (n = 1500) which was stratified for postal codes. An interactive version of the guideline, which was developed from the Agency for Quality in Medicine (ÄZQ) reflecting the content of the guideline of the German Hypertension Society, was sent to 1500 randomly selected physicians throughout Germany. These two intervention groups received in addition to the guideline also a thank-you letter for participation and a summary of the results of the previous study (HEP I).

The control group (n = 1500) was stratified by postal codes from the study group of HEP I.

### Questionnaire

Within 4–6 months after the intervention a re-evaluation of the guideline knowledge was conducted using a casuistic based questionnaire. The questionnaire was based on the guidelines of the German Hypertension Society and was identical with the HEP I questionnaire, which was previously published [2]. The questionnaire comprises ten questions about diagnostic, therapy and therapy control of arterial hypertension.

### Mailing

The mailing of the questionnaires followed the rules of the total design method by Dillman [3]. The questionnaires were mailed to all participants in October 2004. Two weeks later all participants were reminded by a post card. The post card expressed the gratitude to all participants who had returned the questionnaire and served also as a reminder to the other participants. In December 2004 a questionnaire was sent out to all participants who had not yet answered. A last mailing of the questionnaire took place in January 2005.

### Statistical analysis

For the assessment of guideline knowledge 8 out of 10 questions were analyzed. Two questions were excluded from the final analysis because they were used for general orientation about diagnostic (question 2) and therapeutic (question 5) strategies. For each answer conforming to the guidelines one point was awarded and the result was analyzed univariately. A guideline adequate knowledge was certified with five correct answers, these correct answers had to include the correct definition of arterial hypertension (RR ≥ 140/90 mmHg). In addition at least four questions (> 50%) of the analyzed questions had to be answered correctly. The data where analyzed in an explorative way focussing on the differences between intervention groups and control group and between each of the participating physician groups. P < 0.05 was considered statistically significant.

## Results

### Response Rate

Out of a corrected random sample of 4275 addresses 2474 questionnaires could be analyzed which was an effective response rate of 57.9%. The 225 failures of the initial random sample were two physicians who had died, 32 physicians who did not treat patients with hypertension, 50 physicians who had closed their private practice and 141 addresses could not be located by postal means.

After the first mailing process 1114 evaluable questionnaires were sent back corresponding to a return rate of 26.1% of the cleared sample. After the mailing of the post card as a first reminder 219 participants (5.1% of cleared sample) sent back the questionnaire. The second and third reminder (mailing of an additional copy of the questionnaire) were followed by 770 evaluable questionnaires (18%) and finally 371 questionnaires (8.7%).

The return quote for the intervention group with the printed summary of the guideline was 54.9% (n = 783) and for the group with the interactive version of the guideline 59.8% (n = 852). The control group had a return quote of 839 (58.9%) evaluable questionnaires (see Table 1).

There were no relevant differences in demographic data and physician data for responders and non responders to the questionnaire as shown in Table 2.

**Table 2:** Demographic data for study population (cleared sample), responders and non responders.

	**Cleared sample (n = 4275)**	**Responders (n = 2474)**	**Non responders (n = 1801)**
**Female**	26,2%	26,7%	25,4%
**General practice**	37,6%	37,3%	37,9%
**Internal medicine**	57,2%	58,0%	56,1%
**Cardiology**	5,2%	4,7%	6,0%
**Private practice < 5 years**	6,0%	5,8%	6,3%
**Private practice > 20 years**	34,1%	34,4%	33,8%

### Intervention group seminars

Only 2% of the invited physicians in Bremen (invited n = 830) and Karlsruhe (invited n = 732) participated in the seminar. Because of this marginal feedback this intervention arm was cancelled as no meaningful interpretation for this intervention group was possible. Thus, no re-evaluation of the guideline knowledge was performed in this intervention group.

### Guideline awareness in relation to intervention groups

**Figure 1:**
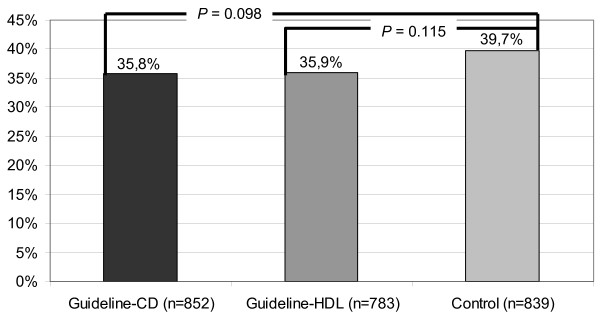
Guideline awareness in relation to intervention groups (n = 2474).

The mailing of printed or interactive guidelines did not improve the adequate knowledge of guidelines. The proportion of physicians with adequate knowledge of guidelines was in the intervention groups even a little less than in the control group, however there was no significant difference (P = 0.098 and P = 0.115) (see Figure 1).

### Guideline awareness in relation to intervention groups and professional specification

**Figure 2:**
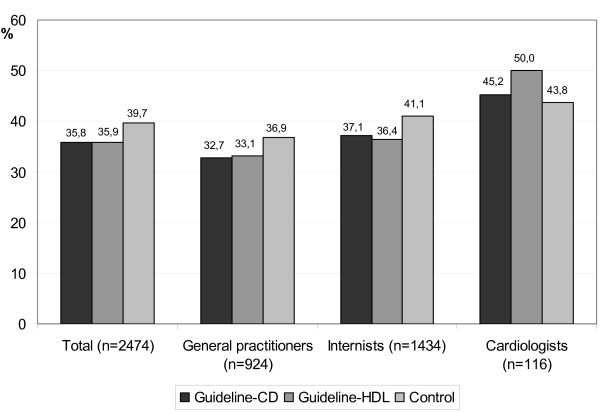
Guideline awareness in relation to intervention groups and professional specification (*P *= n.s.).

The guideline knowledge in the intervention groups differentiated by professional specification showed for general physicians and internists lower proportion of physicians with adequate guideline knowledge than in the control group. Among cardiologists the intervention group with printed guideline was first with adequate guideline knowledge of 50%. The group with the guideline CD was second (45.2%) compared with a control group with 43.8% (see Figure 2).

### Guideline awareness in relation to professional specification

**Figure 3:**
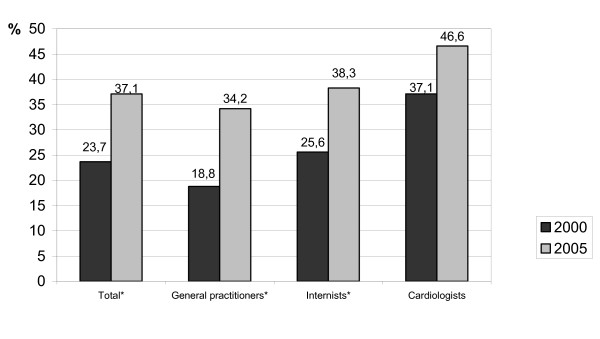
Guideline awareness in relation to professional specification in 2000 (n = 11547) and 2005 (n = 2474) for the total sample. * *P *< 0.0001 for comparison of 2000 and 2005.

The proportion of physicians with adequate guideline knowledge was 37.1% in the total population (n = 919/2474). Among the cardiologists there were 46.5% (n = 54/116), within the internists 38.3% (n = 549/1434) and within general practitioners 34.2% (n = 316/924). In the total population as well as in the subgroups of general practitioners and internists there was a highly significant improvement of the knowledge of guideline contents compared to the HEP I data from the year 2000 (p < 0.001). In contrast there was only a nearly significant improvement in the subgroup of cardiologists (37.1% in 2000 to 46.6% in 2005, p = 0.055). These results are shown in Figure 3 comparing the current data with data from HEP I in 2000.

### Guideline awareness in relation to duration of private practice

**Figure 4:**
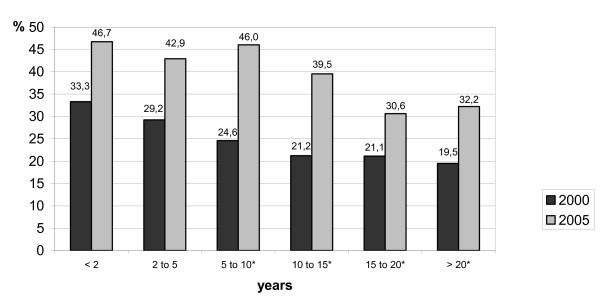
Guideline awareness in relation to duration of private practice in 2000 (n = 11547) und 2005 (n = 2474). * *P *< 0.0001 for comparison of 2000 and 2005.

The proportion of physicians with adequate knowledge of guidelines decreased with the duration of working in private practice. For physicians working in private practice for less than two years almost every second physician showed an adequate knowledge of guidelines. In contrast, in physicians working in private practice for more than 15 years less than every third physician showed an adequate knowledge of guidelines. The results in comparison with the HEP I trial are shown in Figure 4.

Within the subgroup of physicians with guideline adequate definition of arterial hypertension there was an adequate knowledge of guidelines in 68.6% (919/1339). This finding is slightly better than the result from HEP I (66.8%). For general practitioners this figure is 69.0% (310/458), for the internists 67.9% (549/808) and for the cardiologists 74.0% (53/73).

The complete data for the response rate, the adequate knowledge of guidelines for the total population and for the subgroups of physicians with guideline adequate definition of arterial hypertension and adequate knowledge of guidelines with respect to duration of private practice are displayed in Table 3. The group of physicians with duration of private practice of less then 5 years is small as the current data are based on a longitudinal evaluation. The physicians of this group had taken over the practice from physicians from the first study population in 2000.

**Table 3:** Complete data for response rate, guideline awareness and duration of private practice.

	**Total**	**General practice**	**Internal medicine**	**Cardiology**
Total sample	4500	1693	2572	235
Cleared sample	4275	1607	2444	224
Overall response rate	2474	924	1434	116
(%)	57.9	57.5	58.7	51.8
First mailing	1114	404	653	57
First reminder	219	85	125	9
Second reminder	770	309	437	24
Third reminder	371	126	219	26
Adequate guideline awareness	919	316	549	54
(%)	37.1	34.2	38.3	46.6

Subgroup with guideline-conform definition of hypertension ≤ 140/90 mmHg				

Total	1339	458	808	73
Adequate guideline awareness	919	316	549	54
(%)	68.6	69.0	67.9	74.0

Adequate guideline awareness in relation to duration of private practice				

< 2 years	21/45 (46.7%)	4/13 (30.8%)	17/32 (53.1%)	0/0
2–5 years	42/98 (42.9%)	11/35 (31.4%)	28/57 (49.1%)	3/6 (50.0%)
5–10 years	189/408 (46.0%)	40/108 (37.0%)	137/269 (50.9%)	12/31 (38.7%)
10–15 years	269/681 (39.5%)	97/256 (37.9%)	149/377 (39.5%)	23/48 (47.9%)
15–20 years	114/372 (30.6%)	45/158 (28.5%)	63/202 (31.2%)	6/12 (50.0%)
> 20 years	274/850 (32.2%)	114/346 (32.9%)	151/487 (31.0%)	9/17 (52.9%)

## Discussion

These data show that mailing of printed or interactive information had no positive effect on guideline knowledge of physicians. The proportion of physicians with adequate knowledge was even higher in the control group (p = n.s.). Our study corroborates a metaanalysis from Grimshaw [4] for guideline implementation in the Anglo-American area also for Germany.

It is remarkable, that in the intervention group which was invited to a seminar only 2% of the invited physicians finally attended the meeting. Therefore, a continuation of this intervention group was not reasonable. The underlying reason for the modest attendance (lack of top speaker or lack of interest in the topic) can only be assumed.

In spite of the ineffective interventions, overall guideline knowledge improved between 2000 and 2005 from 23.7% to 37.1%. This increase in knowledge was found in all subgroups and was most significant in the group of general practitioners which showed an increase from 18.8% to 34.2%. In the group of cardiologists increase in guideline knowledge was small (37.1% to 46.6%). In summary, the difference between the various physician groups is less pronounced in comparison with the previous investigation in 2000. The deficiency in knowledge depending on the duration of private practice was also less than in the previous study.

The reason for this finding is most likely the combination of time and continuous multimodal interventions. Over a time period of five years there may be various exposures to publications, medical meetings, and internet and so on. This may cause a "contamination" of the study population, but it is thought to affect both, the interventional and control groups. However, our study showed that adding a single intervention over this time period, to this contamination has no additional effect on the guideline awareness. This is similar to the findings of Gross et al. [5] who showed, that single interventions for guideline implementation may sometimes have an effect but are most of the time ineffective. The strategies for implementation of guidelines should be multi-facetted to be effective.

Subgroup analyses of study participants with a guideline adequate definition of arterial hypertension (correct answer to question one: n = 1339) are remarkable. Here we found only a small increase of 0.8% of overall knowledge of guidelines between 2000 and 2005. This validates the knowledge of the correct definition of arterial hypertension as a reliable marker for adequate knowledge of the contents of guidelines and confirms the previous results. Regarding our data, question 1 has a sensitivity of about 70% for adequate knowledge of guidelines.

The unsatisfying level of knowledge about current recommendations for diagnosis and therapy of arterial hypertension partly explains the insufficient control of patients with hypertension in Germany [6]. The HYDRA-Study [7] showed that only 64% of patients with known arterial hypertension in Germany are on treatment and only 19% have a controlled hypertension. In contrast the physicians attested 57% of the patients a well treated hypertension. In the USA 68% of the physicians attested the patients a well treated hypertension [8]. In this study 43% of the patients with hypertension had a blood pressure value of less than 140/90 mmHg.

The MAHLER survey [9] showed for patients with heart failure that a consequent implementation of recommendations of guidelines leads to a significant reduction of hospitalisation.

## Limitations

We had an acceptable response rate of nearly 58% to our questionnaire, but still 42% of the study population (cleared sample) were non responders. Demographic data in Table 2 show that there were no relevant differences between responders and non responders. So it is likely that the results are representative for the total study group.

Over a time period of five years there may be various exposures to publications, medical meetings, and internet and so on. This may cause a "contamination" of the study population, but it is thought to affect both, the interventional and control groups.

## Conclusion

Therefore, the goal must be to improve diagnosis and therapy of arterial hypertension by multimodal interventions. Furthermore there should be additional investigation in regard to the determinants of implementation and medical knowledge to optimize implementation strategies and finally optimize patient outcome.

## Competing interests

The authors declare that they have no competing interests.

## Authors' contributions

All the authors conceived the study and participated in the design. All authors contributed to drafting the manuscript and read and approved the final manuscript.
